# Supplementary figures and images for: Oviductin sets the species-specificity of the mammalian zona pellucida
Source: eLife. 2025 Jun 9;13:RP101338. doi: 10.7554/eLife.101338 (PMC12148327; doi:10.7554/eLife.101338)

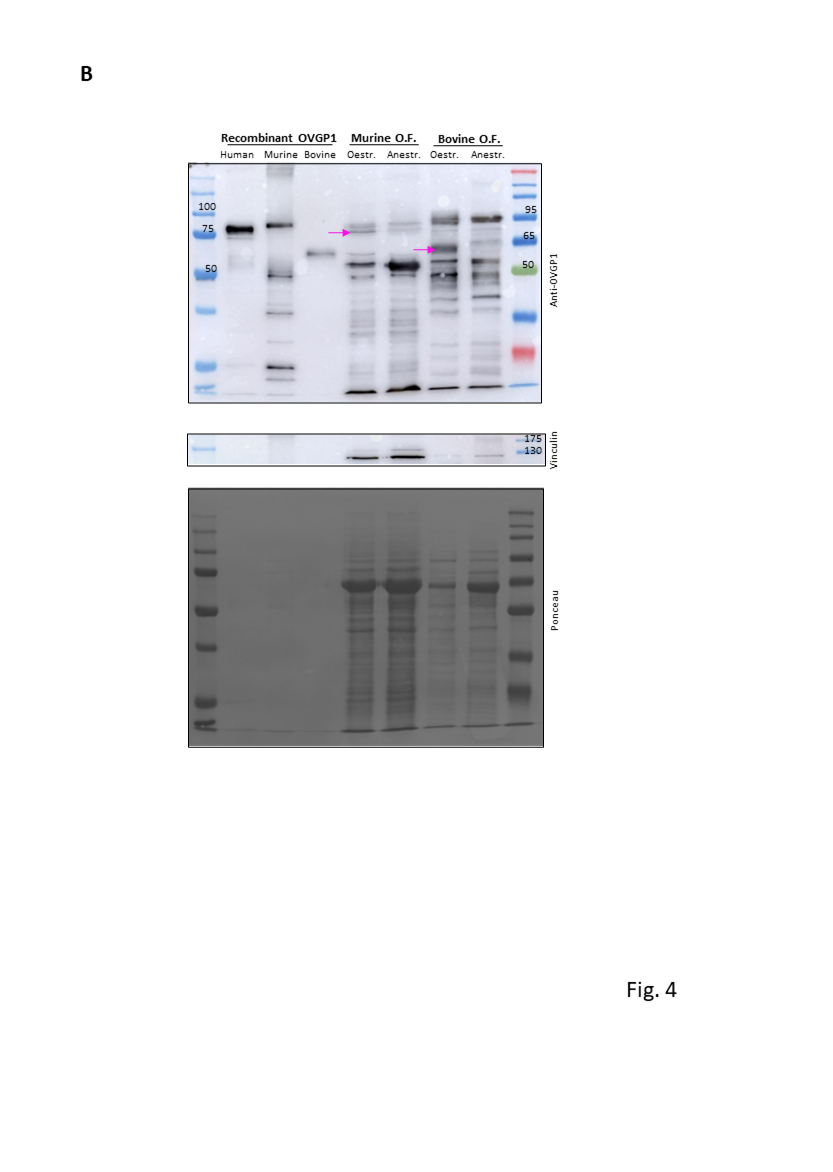

Supplement: Figure 4—source data 1. [file elife-101338-fig4-data1.zip › Fig. 4b WB source data.tif]

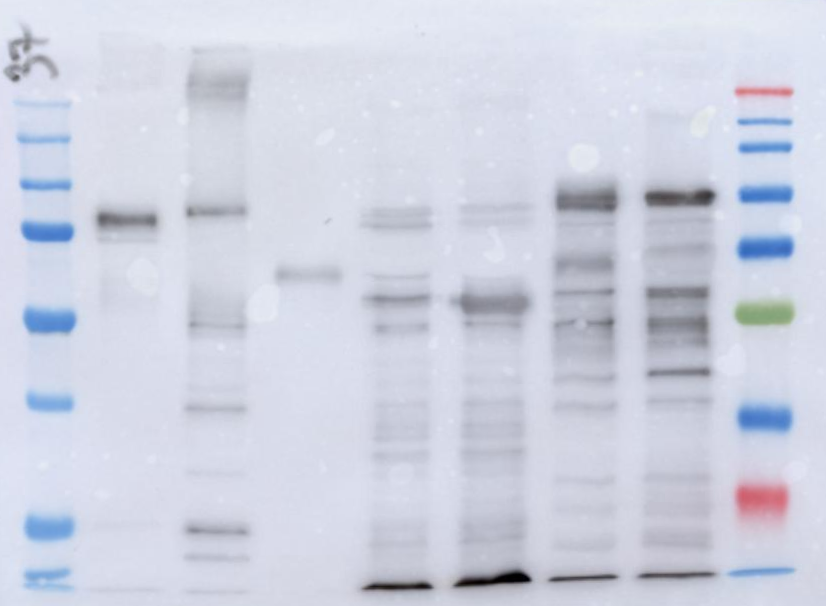

Supplement: Figure 4—source data 2. [file elife-101338-fig4-data2.zip › Fig 4b WB source data Anti-OVGP1.tif]

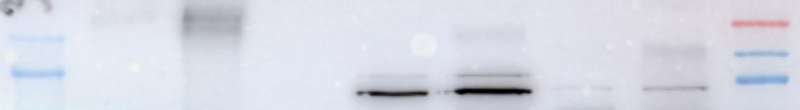

Supplement: Figure 4—source data 2. [file elife-101338-fig4-data2.zip › Fig 4b WB source data anti-Vinculin.tif]

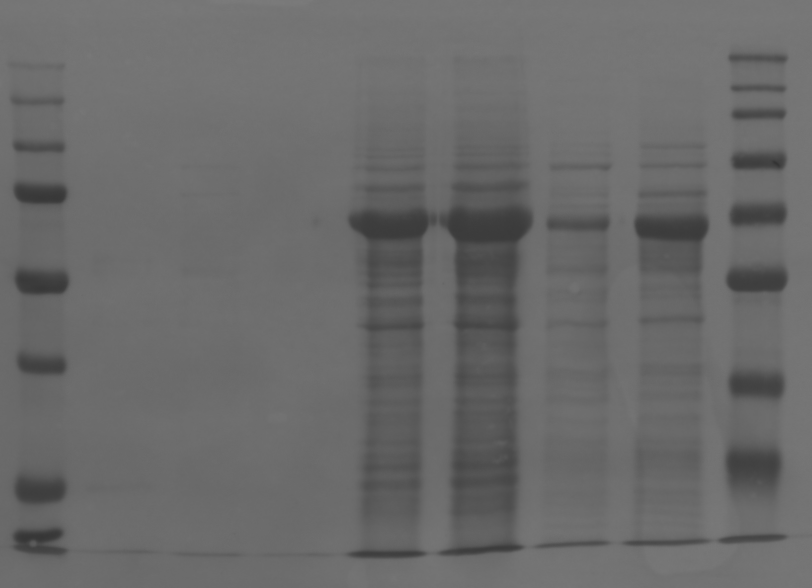

Supplement: Figure 4—source data 2. [file elife-101338-fig4-data2.zip › Fig 4b WB source data Ponceau.tif]

**A**

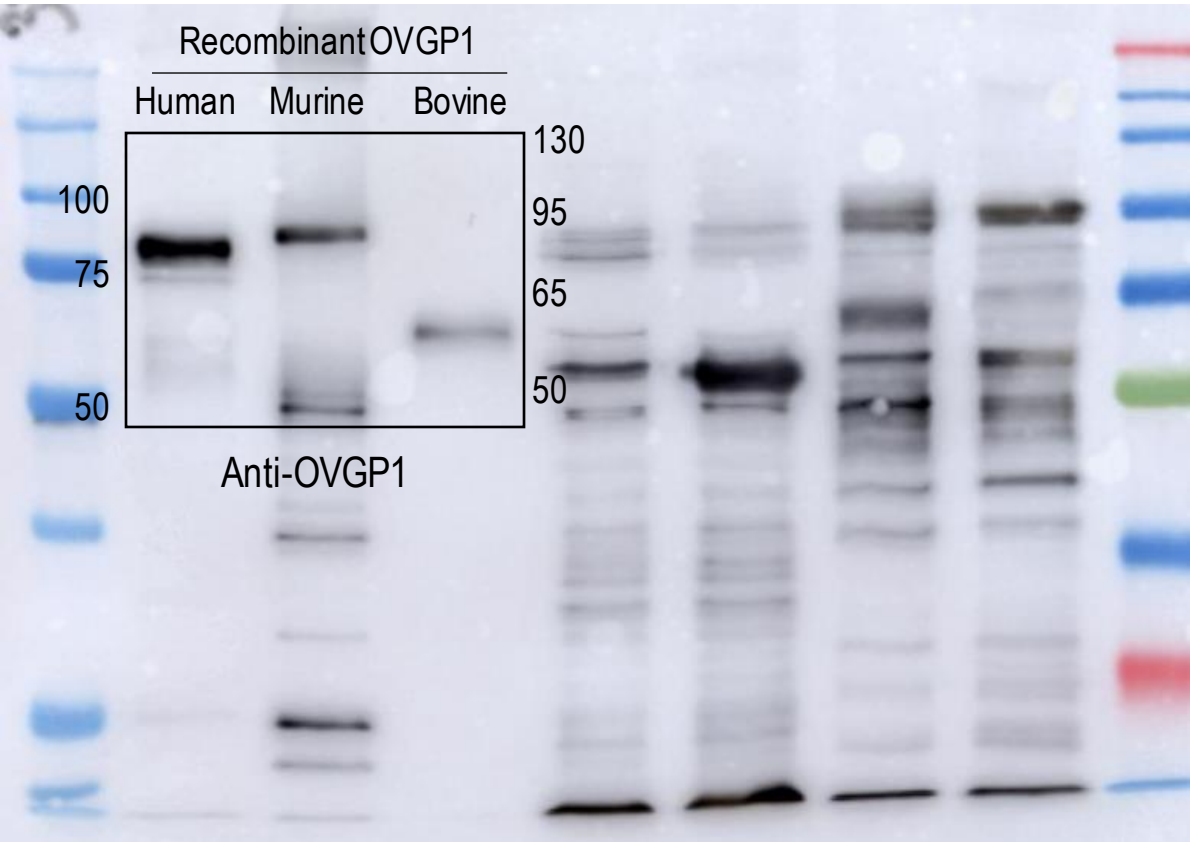

**B**

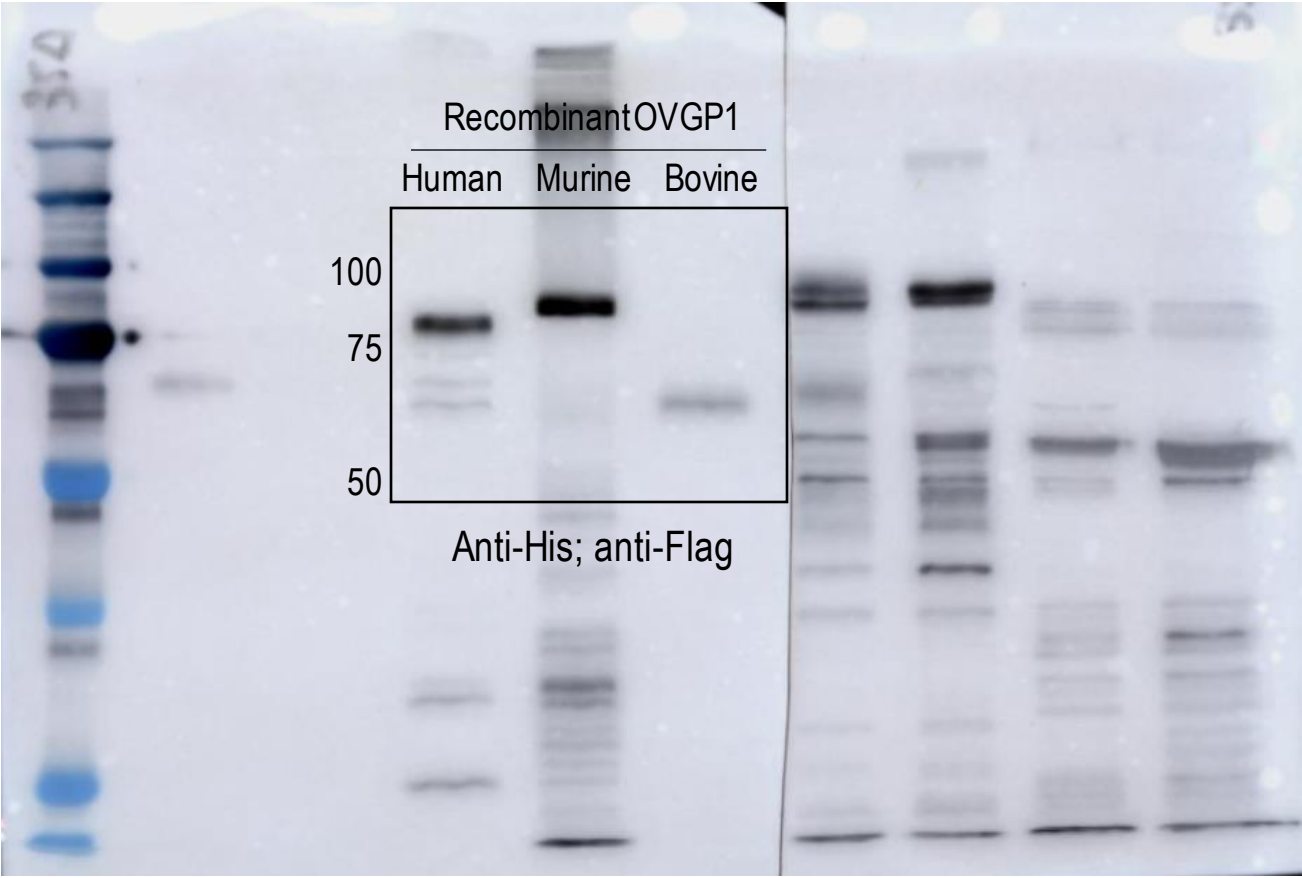

Supplement: Figure 4—figure supplement 2—source data 1. [file elife-101338-fig4-figsupp2-data1.zip › Fig. Supplem WB sin recortar editada.pdf]

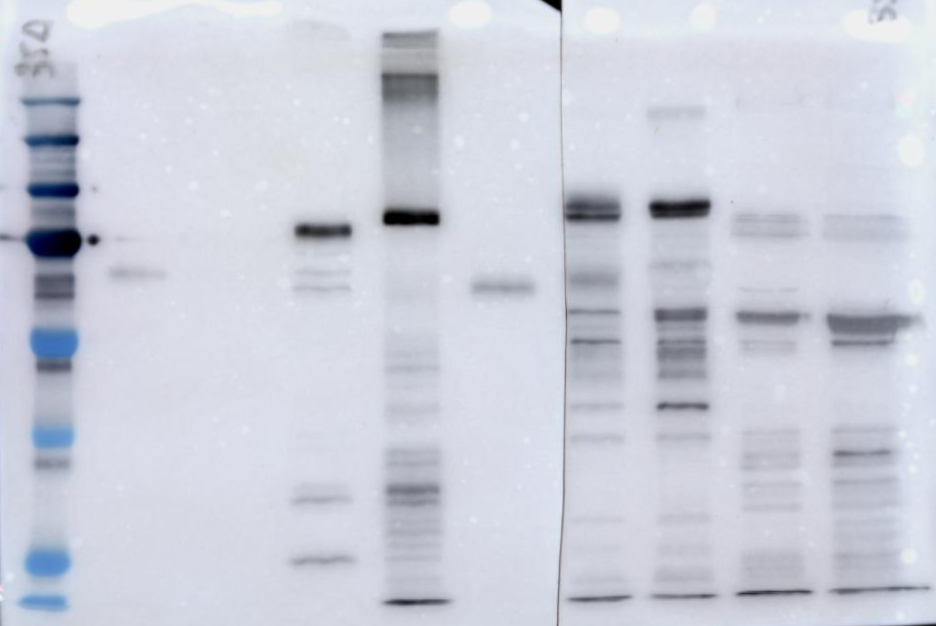

Supplement: Figure 4—figure supplement 2—source data 2. [file elife-101338-fig4-figsupp2-data2.zip › Fig Supplem WB source data anti-His and anti-Flag.tif]
