# Supplementary material for: Oviductin sets the species-specificity of the mammalian zona pellucida
Source: eLife. 2025 Jun 9;13:RP101338. doi: 10.7554/eLife.101338 (PMC12148327; doi:10.7554/eLife.101338)
Supplement: MDAR checklist [file elife-101338-mdarchecklist1.docx]

**Materials Design Analysis Reporting (MDAR)**

**Checklist for Authors**

The [MDAR framework](https://osf.io/xfpn4/) establishes a minimum set of requirements in transparent reporting mainly applicable to studies in the life sciences.

*eLife* asks authors to **provide detailed information within their article** to facilitate the interpretation and replication of their work. Authors can also upload supporting materials to comply with relevant reporting guidelines for health-related research (see [EQUATOR Network](http://www.equator-network.org/%20)), life science research (see the [BioSharing Information Resource](http://biosharing.org/)), or animal research (see the [ARRIVE Guidelines](http://www.plosbiology.org/article/info:doi/10.1371/journal.pbio.1000412) and the [STRANGE Framework](https://doi.org/10.1038/d41586-020-01751-5); for details, see *eLife*’s [Journal Policies](https://reviewer.elifesciences.org/author-guide/journal-policies)). Where applicable, authors should refer to any relevant reporting standards materials in this form.

For all that apply, please note **where in the article** the information is provided. Please note that we also collect information about data availability and ethics in the submission form.

**Materials:**

| **Newly created materials** | **Indicate where provided: section/figure legend** | **N/A** |
| --- | --- | --- |
| The manuscript includes a dedicated "materials availability statement" providing transparent disclosure about availability of newly created materials including details on how materials can be accessed and describing any restrictions on access.  1) Zona pellucidae production from bovine and murine oocytes  2) Bovine, human and mouse OVGP1 recombinant protein | 1) Section Materials and Methods, Preparation of empty zona pellucidae from bovine ovarian oocytes and Preparation of empty zona pellucidae from bovine ovarian oocytes, Figure 2-figure supplement 4  2) Section Materials and Methods, Origins of bovine, murine and human OVGP1 recombinants and Figure 4 |  |
|  |  |  |
| **Antibodies** | **Indicate where provided: section/figure legend** | **N/A** |
| For commercial reagents, provide supplier name, catalogue number and [RRID](https://scicrunch.org/resources), if available.  1) Anti-OVGP1 rabbit polyclonal antibody (NBP1-76939) (Novus Cat# NBP1-76939, RRID:AB_11040013)  2) Anti-Histidine Tag antibody rabbit monoclonal clone RM146 (SAB5600227, Sigma-Aldrich)  3) Anti- Flag M2 antibody mouse monoclonal 639 (F1804, Sigma-Aldrich (Sigma-Aldrich Cat# F1804, RRID:AB_262044)  4) Secondary antibody goat anti-rabbit 693 IgG-HRP (Cat. No. GTX213110-01, GeneTex) (GeneTex Cat# GTX213110-01, RRID:AB_10618573)  5) Secondary antibody goat anti-mouse mIgGk-HRP (sc.516102, 694 Santa Cruz) (Santa Cruz Biotechnology Cat# sc-516102, RRID:AB_2687626)  6) Secondary antibody Alexa Fluor 647 goat anti-rabbit (H+L)  723 (A21245, Invitrogen) (Thermo Fisher Scientific Cat# A-21245, RRID:AB_2535813)  7) Secondary antibody Alexa Fluor 488 goat anti-mouse IgG (H+L) (A11029, Invitrogen) (Molecular Probes Cat# A-11029, RRID:AB_2534088) | 1-5) Section Materials and Methods, Western blotting of OVGP1 and Figure 4 and Figure 4-figure supplement 2.  1, 2, 3, 6 and 7) Section Materials and Methods, OVGP1 immunofluorescence and Figure 4 |  |
|  |  |  |
| **DNA and RNA sequences** | **Indicate where provided: section/figure legend** | **N/A** |
| Short novel DNA or RNA including primers, probes: Sequences should be included or deposited in a public repository.  1) Expression vector pcDNA3.1was cloned with the coding sequence of bovine OVGP1 sequence and six His-tag  2) pH224 plasmid  3) Human (NM_002557.4) and mouse (NM_007696.2) recombinant oviductin proteins (Origene Technologies Inc.). | 1, 2 and 3) Section Materials and Methods, Origins of bovine, murine and human OVGP1 recombinants |  |
|  |  |  |
| **Cell materials** | **Indicate where provided: section/figure legend** | **N/A** |
| Cell lines: Provide species information, strain. Provide accession number in repository OR supplier name, catalog number, clone number, OR RRID.  1) BHK-21 cells (ATCC CCL-10) | 1) Section Materials and Methods, Origins of bovine, murine and human OVGP1 recombinants |  |
| Primary cultures: Provide species, strain, sex of origin, genetic modification status. |  | **N/A** |
|  |  |  |
| **Experimental animals** | **Indicate where provided: section/figure legend** | **N/A** |
| Laboratory animals or Model organisms: Provide species, strain, sex, age, genetic modification status. Provide accession number in repository OR supplier name, catalog number, clone number, OR RRID.  1) Murine sperm was obtained from B6CBAF1 (C57BL/6xCBA) wild type male mice aged 8-20 weeks.  2) Murine oocytes were obtained from wild-type female mice B6CBAF1 (C57BL/6xCBA) aged 8 to 10 weeks. | 1) Section Materials and Methods,  Sperm collection, cryopreservation, thawing, and capacitation  2) Section Materials and Methods, Collection and in vitro maturation of ovarian cumulus-oocyte complexes in mice |  |
| Animal observed in or captured from the field: Provide species, sex, and age where possible.  Cat spermatozoa were obtained from routine gonadectomies were performed tomcats at partnered veterinary centres. Animal handling complied with Spanish Animal Protection Regulation RD53/2013, which conforms to European Union Regulation 2010/63 | Section Materials and Methods, Sperm collection, cryopreservation, thawing, and capacitation |  |
|  |  |  |
| **Plants and microbes** | **Indicate where provided: section/figure legend** | **N/A** |
| Plants: provide species and strain, ecotype and cultivar where relevant, unique accession number if available, and source (including location for collected wild specimens). |  | **N/A** |
| Microbes: provide species and strain, unique accession number if available, and source.  E. *coli* Competent cells JM109 Single-Use (L2005 Promega) was used to transform a His-tagged OVGP1 coding sequence cloned into a pcDNA3.1 (+). | Section Materials and Methods, Origins of bovine, murine and human OVGP1 recombinants |  |
|  |  |  |
| **Human research participants** | **Indicate where provided: section/figure legend) or state if these demographics were not collected** | **N/A** |
| If collected and within the bounds of privacy constraints report on age, sex, gender and ethnicity for all study participants. | Human sperm samples were collected from three male donors, whose average age was 25,3 years and Caucasian ethnicity |  |

**Design:**

| **Study protocol** | **Indicate where provided: section/figure legend** | **N/A** |
| --- | --- | --- |
| If the study protocol has been pre-registered, provide DOI. For clinical trials, provide the trial registration number OR cite DOI. |  | **N/A** |
|  |  |  |
| **Laboratory protocol** | **Indicate where provided: section/figure legend** | **N/A** |
| Provide DOI OR other citation details if detailed step-by-step protocols are available. |  | **N/A** |
|  |  |  |
| **Experimental study design (statistics details) *** | | |
| **For in vivo studies: State whether and how the following have been done** | **Indicate where provided: section/figure legend. If it could have been done, but was not, write “not done”** | **N/A** |
| Sample size determination |  | **N/A** |
| Randomisation |  | **N/A** |
| Blinding |  | **N/A** |
| Inclusion/exclusion criteria |  | **N/A** |
|  |  |  |
| **Sample definition and in-laboratory replication** | **Indicate where provided: section/figure legend** | **N/A** |
| State number of times the experiment was replicated in the laboratory.  1) IVF of bovine oocytes were performed three times for each group  2) EZPT of bovine and murine ZPs were performed three times for each group  3) Bovine and murine oviductal fluids were collected three times. Each lane of the Western Blot represents the oviductal fluid of a cow and five female mice.  4) Western blots were repeated three times, as biological and technical replicates for oviductal fluids and technical replicates for recombinant proteins  5) Immunofluorescence experiments performed with biological and technical replicates  6) Proteomics experiment and analysis was performed once, without replication  7) Electron microscopy experiment and analysis was performed with biological and technical replicates  8) Neuraminidase incubation was replicated three times, technical and biological replicates | 1) Section Materials and Methods, Homologous and heterologous IVF of bovine oocytes  2) Section Materials and Methods, Empty zona penetration test (EZPT) of murine and bovine zona pellucidas  3) Section Materials and Methods, Collection of oviductal fluid, and Figure 4 B  4) Section Materials and Methods, Western blotting of OVGP1  5) Section Materials and Methods, OVGP1 immunofluorescence  6) Section Materials and Methods, Proteomics identification of murine OVGP1  7) Section Materials and Methods, Scanning Electron Microscopy and Image Processing  8) Section Materials and Methods, Incubation of ZP, OVGP1, and sperm with neuraminidase (NMase) |  |
| Define whether data describe technical or biological replicates. |  | **N/A** |
|  |  |  |
| **Ethics** | **Indicate where provided: section/submission form** | **N/A** |
| Studies involving human participants: State details of authority granting ethics approval (IRB or equivalent committee(s), provide reference number for approval.  Approval for the study protocol was obtained from the Hospital Clinico San Carlos Research Ethics Review Committee (Madrid, Spain) and the FivCenter fertility clinic (Aravaca, Madrid, Spain) in accordance with the principles of the Declaration of Helsinki | Section: Material and methods: Sperm collection, cryopreservation, thawing, and capacitation  REFERENCE NUMBER: C.P. IND2022/BIO23646 - C.I. 23/428-E |  |
| Studies involving experimental animals: State details of authority granting ethics approval (IRB or equivalent committee(s), provide reference number for approval.  Ethical aspects related to obtaining sperm from animals and obtaining oocytes. | Section: Material and methods |  |
| Studies involving specimen and field samples: State if relevant permits obtained, provide details of authority approving study; if none were required, explain why. |  | **N/A** |
|  |  |  |
| **Dual Use Research of Concern (DURC)** | **Indicate where provided: section/submission form** | **N/A** |
| If study is subject to dual use research of concern regulations, state the authority granting approval and reference number for the regulatory approval. |  | **N/A** |

**Analysis:**

| **Attrition** | **Indicate where provided: section/figure legend** | **N/A** |
| --- | --- | --- |
| Describe whether exclusion criteria were pre-established. Report if sample or data points were omitted from analysis. If yes, report if this was due to attrition or intentional exclusion and provide justification. | Material and methods section. Only normozoospermic donors were included | **N/A** |
|  |  |  |
| **Statistics** | **Indicate where provided: section/figure legend** | **N/A** |
| Describe statistical tests used and justify choice of tests.  Results are provided as means ± standard deviation (SD). Means were compared and analyzed by repeated measures one-way analysis of variance (ANOVA), followed by Tukey's post hoc test. Significance was set at p < 0.05. | Section: Statistical analysis  Figures: 2, 3, 5, 7, 8, 9. |  |
|  |  |  |
| **Data availability** | **Indicate where provided: section/submission form** | **N/A** |
| For newly created and reused datasets, the manuscript includes a data availability statement that provides details for access (or notes restrictions on access). |  | **N/A** |
| When newly created datasets are publicly available, provide accession number in repository OR DOI and licensing details where available. |  | **N/A** |
| If reused data is publicly available provide accession number in repository OR DOI, OR URL, OR citation. |  | **N/A** |
|  |  |  |
| **Code availability** | **Indicate where provided: section/figure legend** | **N/A** |
| For any computer code/software/mathematical algorithms essential for replicating the main findings of the study, whether newly generated or re-used, the manuscript includes a data availability statement that provides details for access or notes restrictions. |  | **N/A** |
| Where newly generated code is publicly available, provide accession number in repository, OR DOI OR URL and licensing details where available. State any restrictions on code availability or accessibility. |  | **N/A** |
| If reused code is publicly available provide accession number in repository OR DOI OR URL, OR citation. |  | **N/A** |

**Reporting:**

The MDAR framework recommends adoption of discipline-specific guidelines, established and endorsed through community initiatives.

| **Adherence to community standards** | **Indicate where provided: section/figure legend** | **N/A** |
| --- | --- | --- |
| State if relevant guidelines (e.g., ICMJE, MIBBI, ARRIVE, STRANGE) have been followed, and whether a checklist (e.g., CONSORT, PRISMA, ARRIVE) is provided with the manuscript. |  | **N/A** |

* We provide the following guidance regarding transparent reporting and statistics; we also refer authors to [Ten common statistical mistakes to watch out for when writing or reviewing a manuscript](https://doi.org/10.7554/eLife.48175).

**Sample-size estimation**

- You should state whether an appropriate sample size was computed when the study was being designed
- You should state the statistical method of sample size computation and any required assumptions
- If no explicit power analysis was used, you should describe how you decided what sample (replicate) size (number) to use

**Replicates**

- You should report how often each experiment was performed
- You should include a definition of biological versus technical replication
- The data obtained should be provided and sufficient information should be provided to indicate the number of independent biological and/or technical replicates
- If you encountered any outliers, you should describe how these were handled
- Criteria for exclusion/inclusion of data should be clearly stated
- High-throughput sequence data should be uploaded before submission, with a private link for reviewers provided (these are available from both GEO and ArrayExpress)

**Statistical reporting**

- Statistical analysis methods should be described and justified
- Raw data should be presented in figures whenever informative to do so (typically when N per group is less than 10)
- For each experiment, you should identify the statistical tests used, exact values of N, definitions of center, methods of multiple test correction, and dispersion and precision measures (e.g., mean, median, SD, SEM, confidence intervals; and, for the major substantive results, a measure of effect size (e.g., Pearson's r, Cohen's d)
- Report exact p-values wherever possible alongside the summary statistics and 95% confidence intervals. These should be reported for all key questions and not only when the p-value is less than 0.05.

**Group allocation**

- Indicate how samples were allocated into experimental groups (in the case of clinical studies, please specify allocation to treatment method); if randomization was used, please also state if restricted randomization was applied
- Indicate if masking was used during group allocation, data collection and/or data analysis
